# Machine Learning for *In Silico* Virtual Screening and Chemical Genomics: New Strategies

**DOI:** 10.2174/138620708785739899

**Published:** 2008-09

**Authors:** Jean-Philippe Vert, Laurent Jacob

**Affiliations:** Centre for Computational Biology, Mines ParisTech, 35 rue, Saint-Honoré, 77300 France; Institut Curie, Paris, 75248 France; INSERM, U900, Paris, F-75248 France

## Abstract

Support vector machines and kernel methods belong to the same class of machine learning algorithms that has recently become prominent in both computational biology and chemistry, although both fields have largely ignored each other. These methods are based on a sound mathematical and computationally efficient framework that implicitly embeds the data of interest, respectively proteins and small molecules, in high-dimensional feature spaces where various classification or regression tasks can be performed with linear algorithms. In this review, we present the main ideas underlying these approaches, survey how both the “biological” and the “chemical” spaces have been separately constructed using the same mathematical framework and tricks, and suggest different avenues to unify both spaces for the purpose of *in silico *chemogenomics.

## 1 INTRODUCTION

Recent years have witnessed impressive progress in computational biology and chemistry, due in large part to the formulation of many problems as classification or regression problems and to the use of modern machine learning techniques for the analysis of biological and chemical data. In computational biology, typical problems include the prediction of various structural or functional properties of proteins from the description of their amino acid sequence, 3D structure or level of expression in various experimental conditions [1-4], while in computational chemistry the objects manipulated are rather small molecules and the challenges are to predict various chemical or biological properties of these molecules related to their toxicity, pharmacokinetics or affinity for a drug target [5-7]. Both fields are confronted with similar challenges, including the difficulty to manipulate complex and structured data such as molecules, the computational and statistical burden to deal with large-dimensional representations of these data, and the need to integrate heterogeneous description of the same objects, such as the 2D and the 3D structures of molecules.

Among the variety of tools available in statistics and machine learning [8-10], a recent class of algorithms, called *kernel methods*, lends itself particularly well to the study of these aspects [11-13]. The basic philosophy of kernel methods is that with the use of a certain type of similarity measure, called a *kernel*, the data are implicitly embedded in a possibly high-dimensional vector space, in which linear methods are used for various applications such as discrimination, regression, ranking, outlier detection or low-dimensional visualization. The best-known kernel method is the support vector machine (SVM) [14-16], which has quickly gained popularity among the research communitiesfor its efficiency and relative ease of use, and can nowadays be considered as a standard algorithm for regression and supervised classification in both computational biology and chemistry [11, 17, 18].

Choosing a kernel similarity function between proteins or small molecules is a prerequisite to the use of kernel methods, akin to choosing a set of descriptors to represent them. In fact, the inner product or Tanimoto coefficient between fingerprints of descriptors, widely used in chemoinformatics to assess the similarity of small molecules, are valid kernels that can be directly used by kernel methods. In that case, the SVM can be considered as just another multivariate method for regression or classification, that can be used in place of other methods such as partial least squares (PLS) or artificial neural networks (ANN). Interestingly, kernels can also be extended to more general measures of similarity, involving for example an infinite number of descriptors. In such cases, computational tricks are needed to compute the kernel without explicitly computing nor storing the infinitely many descriptors. This extension allows kernel methods to perform multivariate analysis in infinite dimension, often at no additional computational cost compared to the finite-dimensional setting. This interesting property has been investigated by a number of researchers recently, who have proposed ingenious kernel similarity functions to encode various properties of proteins and small molecules with a large or infinite number of descriptors [1,19-25]. These kernels have been successfully applied to various problems in computational biology and chemistry, such as gene function classification or toxicity prediction for small molecules.

Although progresses in computational biology and chemistry have largely ignored each other, the new field of chemogenomics is likely to benefit from both fields simultaneously [26-30]. In particular, a central challenge of *in silico *chemogenomics is to develop a coherent framework and computationally efficient algorithms to manipulate data from both the biological and chemical spaces simultaneously. For example, an *in silico *screening of a series of potential ligands against a family of proteins requires to learn a function *f*(*p*,*l*) that predicts whether the ligand *l* can bind to the target protein *p*. In that case the basic objects to be manipulated by machine learning methods are target-ligand pairs (*p*,*l*), instead of individual targets and ligand candidates. We argue in this review that the parallel development of kernel methods in computational biology and chemistry represents a timely and interesting opportunity to provide such a framework. Indeed, both fields have concentrated a lot of energy, through the design of descriptors and kernels, to construct various representations of the biological and chemical spaces, and kernel methods provide systematic tools to combine these different representations into a joint chemical and biological space where *in silico *chemogenomics can take place. Although the idea of creating *ad hoc *representations of target-ligand pairs for the purpose of chemogenomics is not new [31, 32], the use of kernels can provide additional flexibility and computational advantages over the explicit representation of target-ligand pairs as vectors of descriptors, as illustrated by several recent studies [32-36].

We hope that this short review will contribute to the development of further research on the application of kernel methods in chemogenomics. For that purpose, we start by a quick description of the main ideas and definitions underlying kernels and kernel methods in Section 2, before reviewing the recent attempts to design specific kernels for proteins (Section 3) and small molecules (Section 4). Finally, we present recent attempts to adapt kernel methods to chemogenomics in Section 5. 

## 2 KERNELS AND KERNEL METHODS

Many widely-used statistical and machine learning algorithms, including for example PLS or ANN, are designed to manipulate vector data. Using these tools to manipulate and analyze proteins or small molecules therefore poses the problem of representing these data as vectors or, equivalently, defining a set of binary or real-valued descriptors for these data and stacking them to form a vector. The design of molecular descriptors to describe various features of proteins or small molecules has indeed been much investigated over the last decades, and many such descriptors are nowadays routinely used in combination with statistical methods to correlate the structures of molecules with their physicochemical or biological properties. The explicit computation of a finite number* p *of molecular descriptors to represent a molecule* x *by a vector Φ(*x*) = (Φ_1_(*x*),..., Φ_p_(*x*)) nevertheless raises several challenges, including the problem of choosing a set of descriptors sufficiently large to capture the relevant features for a given problem and sufficiently small to allow fast computation and efficient storage.

Kernel methods, including SVM, are a class of algorithms that follow a slightly different strategy to solve the problem of data representation [11-13]. Data do not need to be represented individually as vectors, they need instead to be compared to each other. More formally, instead of converting each protein or small molecule *x* into a *p-*dimensional vector Φ(*x*) for further processing, kernel methods can manipulate data only through a function* k*(*x*,*x*’) that compares any two proteins (or small molecules) *x* and *x*’ and returns a real number. The function* k* is called a *kernel*, hence the name kernel methods. As a result, when a set of *n* proteins (or of *n* small molecules) *x*1,...,*x*n is given as input to a kernel method, the algorithm can only manipulate the data through the *Gram matrix*, which is the square *n* x *n* matrix *K* of all pairwise similarities, whose entry *Ki,j* is equal to *k*(*xi*,*xj*).

Only a certain class of functions *k*, however, can be used in combination with kernel methods. These kernels are often called *positive definite kernels *or more simply *valid kernel*. The technical conditions that a function *k*(*x*,*x*’) must fulfill to be a valid kernel are (i) to be symmetric, i.e., *k*(*x*,*x*’) = *k*(*x*’,*x*) for any pair of data *x* and *x*’, and (ii) to produce only Gram matrices with no negative eigenvalue. Although the second condition can sometimes be difficult to assess for a newly defined function *k*, mathematics textbook abound on examples of valid kernels and on systematic techniques to create them [37-39].

In fact, many valid kernels have been widely used for a long time in chemoinformatics. For example, given any representation of a molecule *x* by a vector of *p* descriptors Φ(*x*), the inner product between two vectors Φ(*x*) and Φ(*x*’) representing two molecules *x *and *x*’ is a valid kernel:

(1) $$\begin{array}{ccc} k\left(x,x^{'}\right)=< & \Phi \left(x\right),\Phi \left(x^{'}\right)>= & \sum_{i=1}^{p}\Phi_{i}\left(x\right),\Phi_{i}\left(x^{'}\right) \end{array}$$

Another valid kernel is the Tanimoto coefficient between vectors of binary descriptors [40], which is commonly used to assess the similarity between molecules. When such kernels are used, the vectors of descriptors Φ(*x*) are explicitly computed prior to the computation of inner products or Tanimoto coefficients, and kernel methods like SVM are not fundamentally different from other methods such as PLS or ANN.

**Fig. (1):**
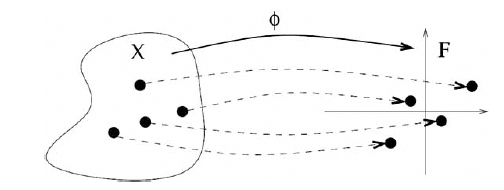
Defining a kernel over a space *X*, such as the space of all small molecules or the space of all proteins, is equivalent to embedding *X* in a vector space *F* of finite or infinite dimension through a mapping Φ:*X → F.*The kernel between two points in *X* is equal to the inner products of their images in *F*, as shown in (1).

Interestingly, it can be shown that, conversely, any valid kernel *k*(*x*, *x*’) can be written as an inner product (1), for some vector representation Φ(*x*) [37]. This apparently establishes an equivalence between the use of valid kernels, on the one hand, and the use of explicit vector representations, on the other hand. In the converse statement, however, the vector Φ(*x*) are not necessarily of finite dimension, they can also involve an infinite number of descriptors. In that case, there is obviously no hope to compute the infinitely many descriptors explicitly and store them in a computer, and a computational trick must be found to compute directly the kernel *k*(*x*, *x*’) without computing Φ(*x*) and Φ(*x*’). We review several examples of such kernels in the next two sections. As a result, the kernel approach can be seen as a generalization of the descriptor vector approach, where the number of descriptors can be finite or infinite (Fig. **1**). Valid kernels therefore always define a vector space structure over the set of molecules to be manipulated.

This structure can either be defined *explicitly*, when molecular descriptors are computed in order to evaluate the kernel similarity through inner products of Tanimoto coefficients between fingerprints, or *implicitly*, when a valid kernel function *k*(*x*,*x*’) is directly computed to compare two molecules x and x. Yet this implicit construction is sufficient to perform various data processing and manipulation in the vector space. As a simple illustration let us consider the problem of computing the distance between two feature vectors Φ(*x*) and Φ(*x*’) corresponding to two molecules *x* and *x*’, as illustrated in Fig. (**2**). A simple computation shows that:

(2) $$\begin{array}{c} \begin{array}{cc} {\left\|\Phi \left(x\right)-\Phi \left(x^{'}\right)\right\|}^{2}= & <\Phi \left(x\right),\Phi \left(x\right)>+<\Phi \left(x^{'}\right),\Phi \left(x^{'}\right)>-2<\Phi \left(x\right),\Phi \left(x^{'}\right)>=k\left(x,x\right)+k\left(x^{'},x^{'}\right)-2k\left(x,x^{'}\right) \end{array} \end{array}$$

where* k *is the kernel associated to the vector Φ through (1). This equation shows that in order to compute the distance between points in the feature space, one does not necessarily need to first compute explicitly the vectors themselves, and can rely instead on the corresponding kernel. This trick, known as the *kernel trick*, can be applied to any algorithm for vectors that can be expressed in terms of inner products: replacing each inner product by the respective kernel evaluation allows the execution of the algorithm implicitly in the feature space defined by a valid kernel. A surprising variety of methods, collectively known as *kernel methods*, can benefit from this trick. Besides the evaluation of distances using (2), one can mention for example dimensionality reduction with principal component analysis (PCA) [41], regression and pattern recognition with Gaussian process [42, 43], PLS [44], SVM [14, 15], or outlier detection with one-class SVM [45]. We refer the reader to various textbooks and survey articles for more details on these algorithms [11-13].

In summary, the definition of a positive definite kernel for certain types of data defines explicitly or implicitly a mapping of the data to a vector space, possibly of high or infinite dimension. Yet a variety of data processing and analysis algorithm can be performed in this feature space thanks to the kernel trick, without the need to compute and store the vector representing the objects. In the next two sections, we review some recent work focusing on the definition of valid kernels for proteins and small molecules, respectively, to illustrate the possibilities offered by kernels to define implicitly “biological” and “chemical” spaces. 

## 3 THE BIOLOGICAL SPACE: KERNELS FOR PROTEINS

Bioinformatics has historically been one of the first application domain for SVM and kernel methods [11], and has triggered a lot of research focused on the design of valid kernels for non-vectorial object, such as proteins. The simplest representation of a protein is the sequence of amino acids it contains, which mathematically is a string in an alphabet of 20 letters. Alternatively, when available or predicted, one can represent a protein by its 3D structure, which is likely to contain more relevant information related to physical interactions with other proteins or ligands. As summarized in Table **1**, three main strategies have been followed to define kernels between proteins: (i) computing an inner product with descriptors explicitly defined, (ii) deriving a kernel from a probabilistic model, and (iii) adapting widely used measures of similarity between biological sequences or 3D structures. We now review in more details these different strategies, starting with kernels defined for amino acid or nucleotide sequences.

The first strategy to make a kernel is to define a set of descriptors to characterize various features of protein sequences, and to compute the inner products between the resulting vectors to obtain a kernel. As an example, Leslie *et al.* [48] uses as descriptors how many times each sequence of *n* letters occurs consecutively in the string, for a fixed integer n (a sequence of *n *contiguous letters is called a *n*-mer). These descriptors could be relevant to detect homologous proteins, which are likely to contain similar proportions of the various *n-*mers, or to predict biological properties that depend on short motifs of amino acids, such as structural or binding motifs. Taking for instance *n* = 2, the DNA sequence* x = AATCGCAACT* is represented by the 16-dimensional vector Φ(*x*) = (2, 1, 0, 1, 1, 0, 1, 1, 0, 1, 0, 0, 1, 0, 0), where the numbers are the counts of occurrences of each 2-mer *AA*, *AC*, . . ., *TG*, *TT* lexicographically ordered. The dimension of Φ(*x*) is then 4^*n*^ for nucleotide sequences, and 20^*n*^ for amino acid sequences, which can be prohibitively large for, e.g., *n* = 5. Fortunately, Leslie *et al. *[48] show that a computational trick allows to compute the kernel between two sequences with a complexity in time and memory linear with respect to the sum of the length of the sequences, independently from the dimension of Φ(*x*) [48, 64]. Several variants to this kernel have also been proposed, including kernels based on counts of *n-*mers appearing with up to a few mismatches in the sequences [20], matching of* n*-mers with the possibility of gaps or substitution [49], or counts of *n-*mers derived from a profile instead of a single sequence [50,51]. Alternatively one can first replace each amino acid by one or several numerical features, such as physicochemical properties, and then extract features from the resulting variable-length numerical time series using classical signal processing techniques such as Fourier transforms [46] or autocorrelation analysis [47]. The resulting features can be explicitly computed to form a vector, and any valid kernel for vector can then be used. These descriptors are interesting to encode information about the variations of physico-chemical properties along the sequence, e.g., to detect elements of the secondary structure. Finally, another popular approach to design features and therefore kernels for biological sequences is to “project” them onto a fixed dictionary of sequences or motifs, using classical similarity measures, and to use the resulting vector of similarities as the feature vector. For example, Logan *et al. *[52] represent each sequence by a 10,000-dimensional vector indicating the presence of 10,000 motifs of the BLOCKS database; similarly, Ben-Hur and Brutlag [53] use a vector that indicates the presence or absence of about 500,000 motifs in the eMOTIF database, requiring the use of a tree structure to compute efficiently the kernel without explicitly storing the 500,000 features; and Liao and Noble [54] represent each sequence by a vector of sequence similarities with a fixed set of sequences. The choice of sequences or motifs to be included in the dictionary is crucial and may be problem dependent, as it allows, for example, the extraction of the occurrences of particular functional or structural motifs in the protein sequences.

A second strategy to design kernels for amino acid sequences has been to derive them from probabilistic models. Indeed, before the interest on string kernels grew, a number of ingenious probabilistic models had been defined to represent biological sequences or families of sequences, including for example Markov and hidden Markov models for protein sequences, or stochastic context-free grammars for RNA sequences [65]. Several authors have therefore explored the possibility to use such models to make kernels, starting with the seminal work of Jaakkola *et al. *[1] that introduced the *Fisher kernel*. This kernel uses a parametric probabilistic model to explicitly extract features from each sequence. The features for a sequence *x* are related to the influence of each parameter of the model on the probability of *x*. The resulting vector of features, known as the Fisher score vector in statistics, has a fixed dimension equal to the number of parameters in the model, and therefore provides a principled way to map sequences of different length to a vector of fixed length. The Fisher kernel was generalized by the Tangent of Posterior (TOP) kernel [55]. Intuitively, the descriptors encoded in the Fisher and TOP kernel describe how each individual sequence differs from a model supposed to represent an “average” sequence, and the choice of the model and its parameters influence therefore a lot the kernel. A second line of thought to make a kernel out of a parametric probabilistic model is to use the concept of mutual information (MI) kernels [66]. Contrary to the Fisher kernel, MI kernels do not provide an explicit finitedimensional representation for each sequence. Instead the dimensions of the feature space are indexed by all possible values of the model parameters, and the feature Φ_θ_(*x*) extracted from the sequence *x* for the parameter θ is the probability of *x* under the model *P*_θ_, i.e., Φ_θ_(*x*) = *P*(*x*|θ). The computation of this kernel involves a summation over all parameters, i.e., takes the form:

$\begin{array}{cc} k\left(x,x?\right)= & \int P_{\theta }\left(x\right) \end{array}P_{\theta }\left(x^{'}\right)d\mu \left(\theta \right)$

where *dµ* is a prior distribution on the parameter space. Hence for practical applications one must chose probabilistic models that allow the computation of the above integral. This was carried out by Cuturi and Vert [56] who presented a family of variable-length Markov models for strings and an algorithm to perform the integral over parameters and models at the same time, resulting in a string kernel with linear complexity in time and memory with respect to the total length of the sequences. There exists an information-theoretic interpretation of mutual information kernels: they quantify how much information is shared between two sequences, in particular if the knowledge of a sequence can be helpful to compress another one. Finally, a third strategy to derive valid kernels from probabilistic models with latent variables, such as HMM, is to build a *marginalized kernel *[19]. Latent variables in probabilistic models often represent meaningful information, such as the local structure or function of a protein sequence. The basic idea behind marginalized kernel is to first design a kernel over the latent and observed variables, as if the latent ones were observed, and then to take the expectation of the kernel with respect to the conditional distribution of the latent variable given the sequences. As for the MI kernel, this kernel can only be computed for judicious choices of random models. Several beautiful examples of such kernels for various probabilistic models have been worked out, including hidden Markov models for sequences [19, 57] or stochastic context-free grammars for RNA sequences [58].

A third strategy to define a kernel is to go back to the interpretation of kernels as “measure of similarity”, and try to adapt well-known measures of similarities between biological sequences to make valid kernels. This idea was pioneered by Haussler [60] who introduced the concept of *convolution kernels *for structured objects that can be decomposed into subparts, such as sequences that can be decomposed into subsequences concatenated to each other (see also [62]). Convolution kernels offer the possibility to combine several kernels adapted to each subpart of the sequences into a single kernel for the whole sequence. Besides proving the validity of convolution kernels, references Haussler [60] and Watkins [61] give several examples of convolution kernels relevant for biological sequences. This work is extended in references [22, 62] where a valid convolution kernel based on the alignment of two sequences is proposed. This kernel, named *local alignment kernel*, is a close relative of the widely used Smith-Waterman local alignment score [67], and gives excellent results on the problem of detecting remote homologs of proteins. This work was later extended to alignment kernels for sequence profiles [63]. This strategy is particularly relevant when the kernel is used by a SVM to predict a property that is conserved across “similar” sequences. In particular, the local alignment score attempts to quantify a measure of evolutionary distance, and the local alignment kernel is therefore particularly adapted to predict biological properties conserved during evolution.

While kernels for sequences, that implicitly map proteins to a feature space through their primary structure, have by far attracted the largest attention so far, several groups have recently attempted to map protein 3D structures through the construction of kernels between structures. Dobson and Doig [3] explicitly represent each structure by a vector made of carefully chosen features, such as secondary structure content, amino acid propensities, surface properties, etc. Alternatively, Borgwardt *et al. *[59] use a representation based upon walks defined on a graph of secondary structural elements, while Qiu *et al. *[25] show that a kernel derived from a measure of structure superpositions is more efficient to relate the structure of a protein to its function.

These kernels for proteins have been widely applied, often in combination with SVM, to various classification  tasks in computational biology, including for example the prediction of structural or functional classes [1, 3, 25, 59, 62, 68-70] or the prediction of the subcellular localization of proteins [4, 71, 72]. The performance reported in these studies is often state-of-the-art, which might be in large part due to the efficacy of algorithms like SVM to estimate classification or regression function. While each kernel for proteins corresponds to a particular embedding of the space of proteins in a vector space, it has been observed that the choice of the kernel, hence of the embedding, can have an important effect on the final performance of the algorithm. For example, in the context of remote protein homology detection, Vert *et al. *[62] compared different kernels and observed that the local alignment kernel was particularly efficient for this application. Besides the performance criterion, different kernels can have different computational complexities which might become prohibitive if large datasets are to be processed. Hence in practical applications the choice of a particular kernel is often a trade-off between computational consideration and performance. 

## 4 THE CHEMICAL SPACE: KERNELS FOR SMALL MOLECULES

Kernel methods are also increasingly used in chemoinformatics for various analysis, regression or classification tasks with small molecules. As for proteins, the design of kernels for small molecules can follow different strategies. We now review the main recent contributions in this field, as summarized in Table **2**.

The problem of explicitly representing and storing small molecules as finite-dimensional vectors has a long history in chemoinformatics, and a multitude of molecular descriptors have been proposed [83]. These descriptors include in particular physicochemical properties of the molecules, such as its solubility or logP, descriptors derived from the 2D structure of the molecule, such as fragment counts or structural fingerprints, or descriptors extracted from the 3D structure. All classical vector fingerprint and vector representations of molecules define an explicit “chemical space” where each molecule is represented by a finite-dimensional vector, and these vector representations can obviously be used as such to define kernels between molecules [73, 74].

Alternatively, several groups have investigated different strategies to build implicit chemical spaces by defining kernels between molecules that do not require the explicit computation of vector representations. These attempts were pioneered simultaneously and independently by Kashima *et al. *[23, 75] and Gartner *et al. *[76] who proposed to represent the 2D structure of a molecule by an infinite-dimensional vector of linear fragment counts and showed how SVM can handle this representation with the kernel trick. Mahé *et al. *[77] extended these works by showing how irrelevant fragments can be filtered out and proposing a trick to increase the dimension of the feature space to make the fragments more specific while simultaneously increasing the speed of the kernel computation. Ralaivola *et al. *[40] also tested several variants of these kernels, and showed in particular that the Tanimoto index, widely used in chemoinformatics, is a valid kernel. Borgwardt and Kriegel [78] investigated the possibility to restrict the fragment counts to shortest-path fragments. Several groups have also tried to extend the substructures extracted from the molecular graphs beyond linear fragments and therefore to trade some increase in expressiveness against loss in computational efficiency [79]. For example, Horvath *et al. *[81] consider kernels based on cyclic fragments, while Ram and Gärtner [79] suggeste that tree fragments be considered instead of linear fragments, an idea that was later extended and validated by Mahé and Vert [80]. Finally, Fröhlich *et al. *[82] defined a kernel between molecular graphs by scoring an optimal matching between the atoms of two molecules to be compared; this kernel, however, is not a valid one [84].

A few attempts to define kernels based on the 3D structure of molecules have also been proposed. Mahé *et al.* [24] design a kernel focused on the detection of 3D pharmacophores, while Swamidass *et al. *[85] consider similarity measures between histograms of pairwise distances between atom classes and Azencott *et al. *[74] use Delaunay tetrahedrization and other techniques from computational geometry to characterize the 3D structures of small molecules and make kernels. Finally, Azencott *et al. *[74] show how kernels can also handle multiple 3D conformations and demonstrates the relevance of this idea on several QSAR experiments.

Although the construction of valid kernels for molecules is a young discipline, it has witnessed impressive progresses in just a few years, triggered by potential application in chemoinformatics and drug design. Large avenues that could be relevant for kernel design remain however largely unexplored, such as the modeling of 3D surfaces, their electrostaticity and polarity, or the dynamics of the structures. We expect fast progresses in this field in the coming years. 

## 5 TOWARDS *IN SILICO *CHEMOGENOMICS WITH KERNELS

Chemogenomics intends to screen the chemical universe, or at least subsets of this universe, against the therapeutic target universe [26-30, 86, 87]. While quantitative structure-activity relationship (QSAR) analysis in chemoinformatics attempt to model the affinity of a family of molecules for a single target protein, chemogenomics can be thought of as a generalization where several targets are screened simultaneously. The screening process for each target can indeed be expected to benefit from the known data for other targets. In particular it is possible to make accurate prediction for targets with few known ligands or even no known ligand if more data is available for targets that are *similar *to the target of interest [30].

The development of *in silico *chemoinformatics also needs tools to go beyond classical QSAR and classification methods. The problem of QSAR can be formulated as the problem of learning a function *f*(*m*) to predict the affinity of a molecule *m* to a given target. The function *f* can for example be a linear function when *m* is represented by a vector Φ(*m*). In the chemogenomics era, however, multiple targets are considered simultaneously and the problem is rather to infer a function *f*(*m*,*p*) to predict the affinity of a molecule m for a target protein p. In order to apply classical classification or regression tools, a vector representation Φ(*m*,*p*) of the *pair *(*m*,*p*) must therefore be chosen. In other words, instead of simply working with the chemical or biological spaces alone, we must now consider their *product space*, i.e., the set of (*m*,*p*) pairs, and design machine learning algorithms in this product space. Several methods, referred to as *target-ligand approaches *in reference [29], have started to emerge to formally combine the compounds and target universes.

We argue that kernel methods offer an attractive framework for *in silico *chemogenomics, seen as the problem of inferring functions in the product chemical and biological space. Indeed, as surveyed in Sections 3 and 4, a variety of kernels capturing various features or proteins and small molecules have already been defined. Each of these kernels is equivalent to an explicit or implicit definition of the chemical or biological space as a possibly high-dimensional vector space. Now let us suppose that in these spaces, a protein *p* is represented by a vector Φ_*P*_(*p*) corresponding to a kernel *k_P_*, while a small molecule *m* is represented by a vector Φ_*M*_(*m*) corresponding to a kernel *k_M_*. Perhaps the most natural way to represent the pair (*p*,*m*) for the purpose of chemogenomics is to concatenate both vectors Φ_*P*_(*p*) and Φ_*M*_(*m*) into a single vector Φ_*M*_(*m*,*p*) [32]. Interestingly, in terms of kernels, it is easy to show that the inner product in the joint space obtained by concatenating the vectors, which is usually called the tensor sum kernel and denoted *k_P_* ⊕ *k_M_*, is simply the sum of the inner products in both spaces taken separately, and therefore that the kernel for (*p*,*m*) is simply the sum of the protein kernel and of the molecule kernel [37]:

$\begin{array}{cc} k_{p}⊕k_{M}\left(p,m\right),\left(p^{,},m^{,}\right)= & k_{p}\left(p,p^{,}\right)+k_{M}\left(m,m^{,}\right) \end{array}$

A drawback of this sum is that if a linear algorithm is run on the concatenation of two vectors, it results in a linear function f(p,m) that decomposes as the sum of a protein-specific function and a molecule-specific function, i.e., *f*(*p*,*m*)= *f_P_*(*p*) + *f_M_*(*m*). In particular, if the function *f*(*p*,*m*) is then used to rank molecules *m* for a fixed protein *p*, then the ranking only depends on the molecule-specific temr *f_M_*(*m*). A undesired consequence is that the ranking of molecules in then the same for all proteins.

More interestingly, correlations between the protein and the molecule descriptors can be introduced by considering, instead of the simple concatenation of vectors, the tensor product between vectors whose descriptors are all possible products between a protein descriptor and a molecule descriptor. As a result, if a protein is characterized by *d_P_* descriptors and a small molecule by *d_M_* descriptors, then their tensor product is characterized by *d_P_* × *d_M_* descriptors. In the case of binary descriptors that describe the presence or absence of particular features in the protein and the small molecule, a descriptor in the tensor product will be 1 if and only if the corresponding descriptors in the protein and in the small molecule are both equal to 1, hence this representation allows to encode the simultaneous presence of particular features in both the protein and the ligand. Unfortunately, this rich representation can hardly be computed for classical vectors of descriptors due to the explosion in the size of the tensor product vector: for example, using a vector of molecular descriptors of size 1024 for small molecules, and representing a protein by the vector of counts of all 2-mers of amino acids in its sequence (*d_P_* = 20 × 20 = 400) results in more that 400 thousands descriptors in the tensor product. The kernel formulation has a very strong advantage here: indeed, it can be shown with classical algebra that the kernel associated with a tensor product, which is usually called the tensor product kernel and denoted *k_P_* ⊕ *k_M_*, is simply the product of individual kernels [37]:

$\begin{array}{cc} k_{p}⊗k_{M}\left(\left(p,m\right),\left(p^{,},m^{,}\right)\right)= & k_{p} \end{array}\left(p,p^{,}\right)k_{M}\left(m,m^{,}\right)$.

This shows that just multiplying any protein kernel with any small molecule kernel results in a valid kernel for protein-ligand pairs, which encodes a rich information about features jointly present in the protein and the molecule.

Recently, this useful property has been investigated by a few authors [33] who applied the tensor product kernel to two vectors of descriptors for proteins and molecules, in order to estimate a model to predict interactions between drug targets and small molecules from a collection of disparate HTS campaigns aimed at screening 24 biological targets. The molecular descriptors ranged from atom frequencies and topological indexes to 3D surface area descriptors, while the protein descriptors encode information about the amino acids in the binding pocket. The study concluded that the tensor product combined with a SVM facilitates the improvement of the prediction accuracy over models trained for each target independently from each other. We [34] followed a similar approach in the context of epitope prediction for multiple MHC-I alleles, *i.e. *interaction between short peptides and MHC-I molecules. In that case, the candidate epitopes (short peptides of 9 amino acids) were described by the amino acids present at each position, while different kernels for MHC-I molecules were tested, including kernels based on the primary sequence of the proteins, and kernels based on the presence of particular amino acids at key residues of the interaction pocket. It was observed that simultaneously learning predictors for the binding of short peptides to different alleles of the MHC-I, using a SVM in the product space defined by a tensor product kernel, resulted in significant improvement over the learning of predictors for different alleles independently. More recently, some attempts to systematically apply this strategy to large classes of therapeutic targets, including enzymes, G-protein coupled receptors (GPCR), and ion channels have been published [35,36]. Combining again various descriptors for small molecules and protein targets with the tensor product kernel, the authors demonstrate that very accurate models for target-ligand interactions can be inferred by sharing data across targets. In particular, they show that their model is able to infer correct ligands even for targets with no known ligand used during the training of SVM. This suggests that this ligand-based procedure could in principle contribute to the prediction of ligands for orphan drug targets, which is of tremendous interest for the pharmaceutical industry. 

## 6 CONCLUSION

Kernel methods have become prominent in both bioinformatics and chemoinformatics. In particular much effort has been spent on the construction of various kernel functions for proteins and small molecules, which capture various structural, evolutionary or physico-chemical properties of these molecules. Kernel methods provide state-of-the-art performance on many protein or small molecule classification or regression tasks, and offer a promising framework for the new field of *in silico *chemogenomics. In particular various operations on kernel functions allow the principled construction of kernels for target-ligand pairs, resulting in the systematic and implicit construction of a combined target-ligand space amenable to various data analysis by kernel methods. Given the large choice of existing protein and molecule kernels, operations on kernels, and kernel methods, we believe the burgeoning field of kernel methods for *in silico *chemogenomics offers a lot of opportunities to accompany the current *in vitro *and *in vivo *chemogenomics wave.

The first attempts to apply these state-of-the-art machine learning algorithms to *in silico *chemogenomics are very encouraging, although it is still a bit early to assess their real impact on the drug discovery process. It is likely that new issues related to the particular data manipulated will arise, such as the fact that the set of known interactions involves only a very small number of molecules and proteins compared to the huge size of the chemical and biological space, and that the negative results are usually not recorded in HTS campaigns although they may be useful to train *in silico *chemogenomics models. These and other challenges now need to be clearly identified and taken into account for the development and improvement of *in silico *models.

## Figures and Tables

**Fig. (2):**
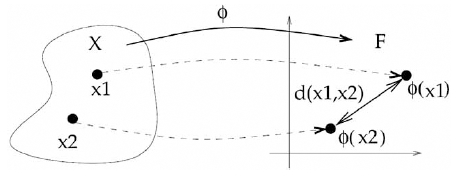
We can define the distance between two objects x1 and x2, such as two small molecules or proteins, as the Euclidean distance between their images Φ(x_1_) and Φ(x_2_). If the mapping Φ is defined by a valid kernel *k*, then this distance can be computed easily without computing Φ(*x*_1_) and Φ(*x*_2_), as shown in (2). This* kernel trick* can be extended to a variety of linear algorithms that only manipulate the data through inner products.

**Table 1:** A Typology of Kernels for Proteins

Strategy	Input Data	Examples
Define a list of descriptors	Sequence	Physico-chemical kernels [46, 47] Spectrum, mismatch kernels [48-51] Pairwise motif kernel [52-54]
	3D Structure	Kernel based on 3D descriptors [3]
Derive a kernel from a generative model	Sequence	Fisher, TOP kernels [1, 55] Mutual information kernels [56] Marginalized kernels [19, 57, 58]
	3D Structure	Random walk kernels [59]
Derive a kernel from a measure of similarity	Sequence	Local alignment kernel [22, 60-63]
	3D Structure	Structure alignment kernel [25]

**Table 2:** A Typology of Kernels for Small Molecules

Strategy	Input Data	Examples
Use classical fingerprints of molecular descriptors	1D to 4D structure	Tanimoto or inner products between fingerprints [40, 73, 74]
Use an infinite number of descriptors and a computational trick	2D structure	Walk kernels [23, 75-77] Shortest-path fragment kernel [78] Subtree kernel [79, 80] Cyclic fragment kernel [81]
	3D Structure	Pharmacophore kernel [24]
Use a measure of similarity	2D structure	Optimal alignment kernel [82]
